# In Silico and In Vitro Analysis of Interaction between Ximelagatran and Human Leukocyte Antigen (HLA)-DRB1*07:01

**DOI:** 10.3390/ijms18040694

**Published:** 2017-03-24

**Authors:** Makoto Hirasawa, Katsunobu Hagihara, Koji Abe, Osamu Ando, Noriaki Hirayama

**Affiliations:** 1 Drug Metabolism & Pharmacokinetics Research Laboratories, Daiichi Sankyo Co., Ltd., 1-2-58 Hiromachi, Shinagawa-ku, Tokyo 140-8710, Japan; hagihara.katsunobu.fc@daiichisankyo.co.jp (K.H.); abe.koji.ce@daiichisankyo.co.jp (K.A.); ando.osamu.jy@daiichisankyo.co.jp (O.A.); 2 Institute of Advanced Biosciences, Tokai University, 4-1-1 Kitakaname, Hiratsuka-shi, Kanagawa 259-1292, Japan; tousa@tsc.u-tokai.ac.jp

**Keywords:** ximelagatran, melagatran, HLA (human leukocyte antigen), hepatotoxicity, IDT (idiosyncratic drug toxicity), MD (molecular dynamics) simulation

## Abstract

Idiosyncratic ximelagatran-induced hepatotoxicity has been reported to be associated with human leukocyte antigen (HLA)-DRB1*07:01 and ximelagatran has been reported to inhibit the binding of the ligand peptide to HLA-DRB1*07:01 in vitro. In order to predict the possible interaction modes of ximelagatran with HLA-DR molecules, in silico docking simulations were performed. Molecular dynamics (MD) simulations were also performed to predict the effect of ximelagatran on the binding mode of the ligand peptide to HLA-DRB1*07:01. A series of in silico simulations supported the inhibitory effect of ximelagatran on the binding of the ligand peptide to HLA-DRB1*07:01 in vitro. Furthermore, direct interactions of ximelagatran with HLA-DR molecules were evaluated in vitro, which supported the simulated interaction mode of ximelagatran with HLA-DRB1*07:01. These results indicated that ximelagatran directly interacts with the peptide binding groove of HLA-DRB1*07:01 and competes with the ligand peptide for the binding site, which could alter the immune response and lead to the idiosyncratic ximelagatran-induced hepatotoxicity.

## 1. Introduction

Idiosyncratic drug toxicity (IDT) is a rare toxic drug reaction characterized by the delayed onset of symptoms, a dose- and duration-independent occurrence, and an unpredictable nature. It is often life-threatening and, thus, one of the major reasons for drug withdrawals or black box warnings Clinical investigations have proposed that the mechanisms of most IDTs are immune-mediated and there is a very strong association with specific human leukocyte antigen (HLA) genes for certain reactions [1].

Ximelagatran (Exanta^®^/Exarta^®^) is an orally-available anticoagulant agent that directly inhibits thrombin and was developed for the prevention and treatment of thromboembolism. Although ximelagatran was generally well tolerated in short-term use (<12 days), an elevated serum alanine aminotransferase (ALT) level of >3× upper limit of normal was found to develop during long-term treatment (>35 days) in 7.9% of patients [2]. Due to the concerns for its potential liver toxicity, the development of ximelagatran was terminated and it was withdrawn from the world market in 2006. A retrospective case-control pharmacogenetic study of ALT elevation during long-term treatment of ximelagatran revealed a strong genetic association between elevated ALT and the HLA alleles DRB1*07 and DQA1*02, suggesting a possible immune-mediated pathogenesis [3]. Furthermore, a competitive binding assay revealed that ximelagatran was able to inhibit the binding of the ligand peptide to HLA-DRB1*07:01, supporting the specific involvement of HLA-DRB1*07:01 in the idiosyncratic ximelagatran-induced hepatotoxicity [3].

**Figure 1:**
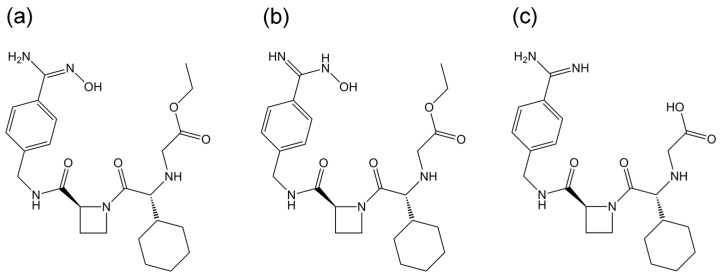
Chemical structures of (**a**) ximelagatran in oxime form; (**b**) ximelagatran in hydroxylamine form; and (**c**) melagatran.

In recent years, the mechanisms of immune stimulation by small molecules without involving covalent binding to macromolecules, such as the “pharmacological interaction with immune receptors” [4,5], and “altered self-repertoire” concepts [6], have been reported. There also have been several reports showing that small molecules can modify the antigen loading onto HLA molecules [5,6,7,8], and the interaction modes of some compounds with HLA molecules have been evaluated by in silico docking simulation [5], MD simulation [9], or X-ray crystalline structure analysis [6]. We previously reported that in vitro lapatinib enhances the binding of the ligand peptide to HLA-DRB1*07:01, which is strongly associated with lapatinib-induced liver injury, and MD simulations indicated the allele specific modification of the structure of the peptide binding groove of HLA-DRB1*07:01 by lapatinib [10]. Ximelagatran also showed idiosyncratic hepatotoxicity in clinical settings, which was strongly associated with the same HLA allele, DRB1*07:01. Interestingly, the same in vitro HLA class II binding assay conducted by EpiVax, Inc. (Providence, RI, USA), resulted in the opposite effects of two drugs on the binding of the same ligand peptide to HLA-DRB1*07:01 [3,10]. Therefore, in the present study, we evaluated the possible interaction modes of ximelagatran and its active metabolite, melagatran (Figure 1) with three HLA-DR molecules in silico to compare them with those of lapatinib. Furthermore, the amount of ximelagatran bound to HLA-DR molecules when it showed the inhibitory effect was evaluated in vitro by liquid chromatography tandem mass spectrometry (LC-MS/MS).

## 2. Results

### 2.1. Docking Simulations

**Figure 2:**
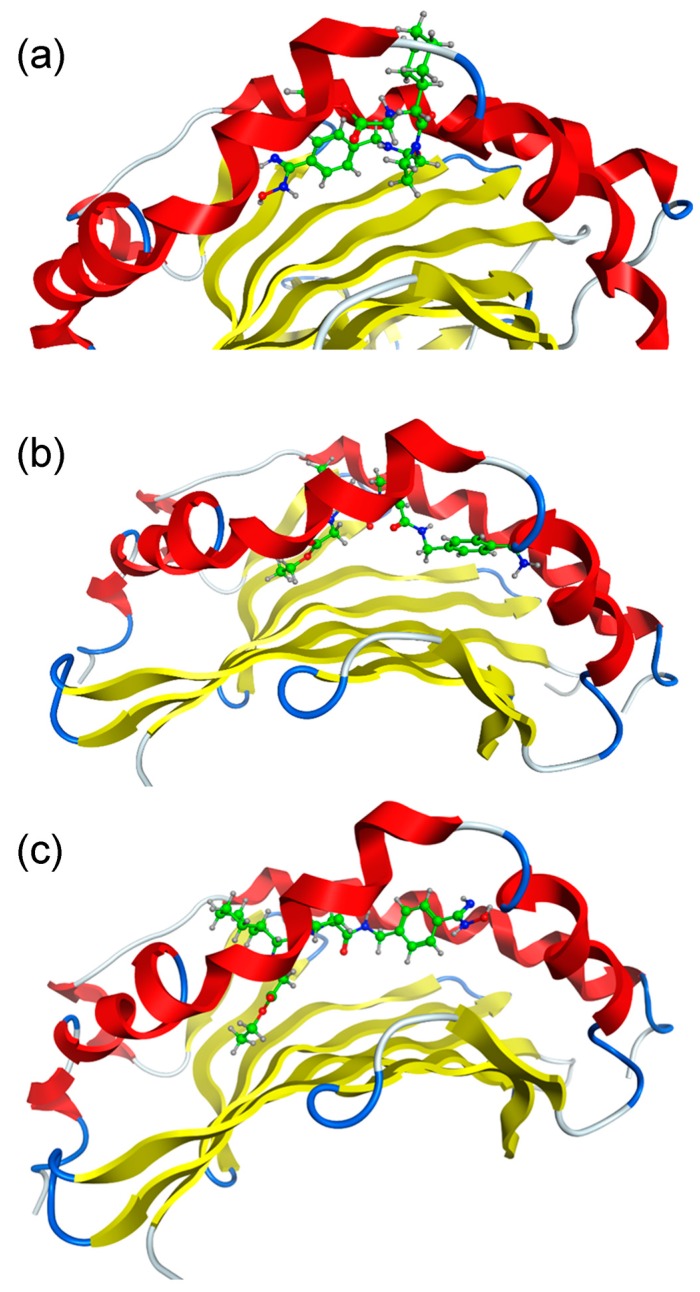
Binding modes of ximelagatran at the peptide binding grooves of human leukocyte antigen (HLA)-DR molecules. (**a**) DRB1*01:01; (**b**) DRB1*07:01; and (**c**) DRB1*15:01. The structures of the HLA-DR molecules are depicted in cartoon mode (α helix in red and β sheet in yellow) and ximelagatran is depicted in a ball-and-stick model (C in green, H in gray, N in blue and O in red).

**Figure 3:**
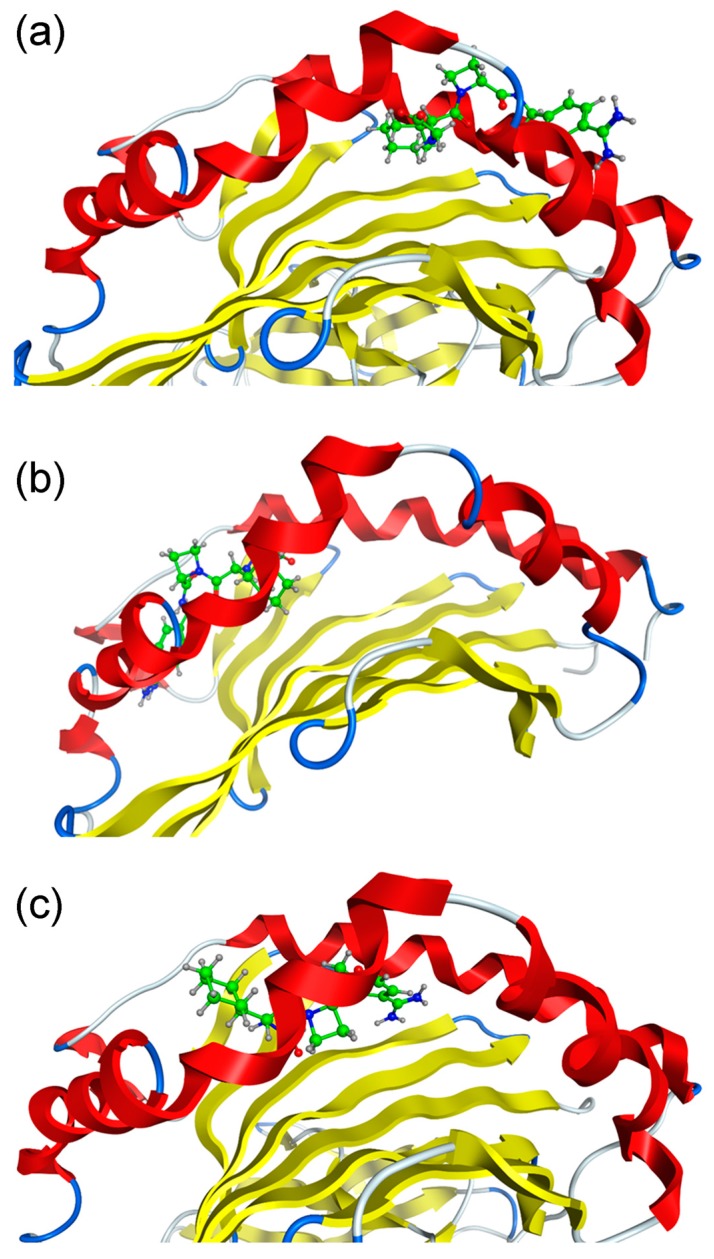
Binding modes of melagatran at the peptide binding grooves of HLA-DR molecules. (**a**) DRB1*01:01; (**b**) DRB1*07:01; and (**c**) DRB1*15:01. The structures of the HLA-DR molecules are depicted in cartoon mode (α helix in red and β sheet in yellow) and melagatran is depicted in a ball-and-stick model (C in green, H in gray, N in blue and O in red).

The interaction modes of ximelagatran or melagatran with HLA-DRB1*07:01 and control alleles DRB1*01:01 and DRB1*15:01, both of which are associated with IDTs caused by other drugs, were predicted by in silico docking stimulations. The binding affinities were judged by a scoring function of generalized-Born volume integral/weighted surface area (GBVI/WSA_dG), which is considered to express protein-ligand binding free energy [11]. The lowest GBVI/WSA_dG values (kcal/mol) of the complexes between ximelagatran or melagatran and the three HLA-DR molecules are shown in Table 1. These values are much lower than those of the complexes between HLA-B*14:02 and nevirapine-related compounds [12], and ones between HLA-B*58:01 and allopurinol-related compounds [13] simulated by the same software system, indicating ximelagatran and melagatran have high potential to interact with these HLA molecules. The binding modes of ximelagatran and melagatran at the peptide binding grooves of HLA-DRB1*01:01, DRB1*07:01, and DRB1*15:01 with the lowest GBVI/WSA_dG values in each complex are shown in Figure 2 and Figure 3, respectively. In the complex with HLA-DRB1*07:01, the oxime form of ximelagatran showed a higher binding affinity than the hydroxylamine form. Ximelagatran showed higher simulated binding affinities than melagatran for all three HLA-DR molecules.

### 2.2. Molecular Dynamics (MD) Simulations

**Figure 4:**
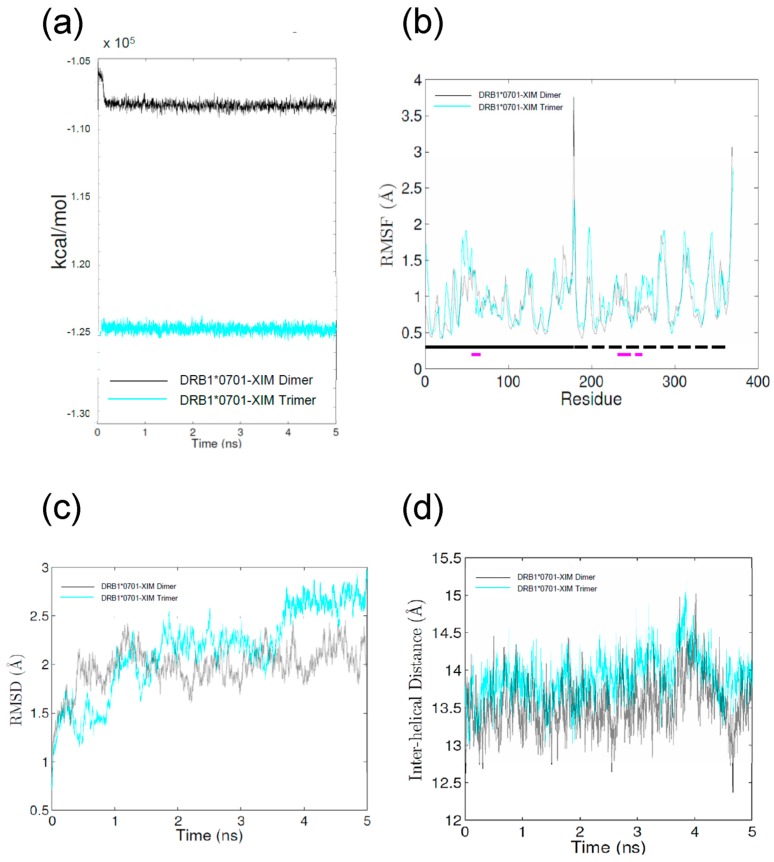
Molecular dynamics (MD) simulation parameters. (**a**) Calculated energies vs. time plot; (**b**) root mean square fluctuation (RMSF) values of polypeptide backbone. The location of α and β chains and the residues that comprise the peptide binding groove helices are indicated by the solid and dashed lines running just above the *x*-axis: α chain (solid black), α chain helix (solid purple), β chain (dashed black), β chain helix (dashed purple); (**c**) RMSD values of the polypeptide backbone vs. time plot; and (**d**) the average distance between each Cα in the helix of the α chain and the closest Cα atom in the helix from the β chain.

**Figure 5:**
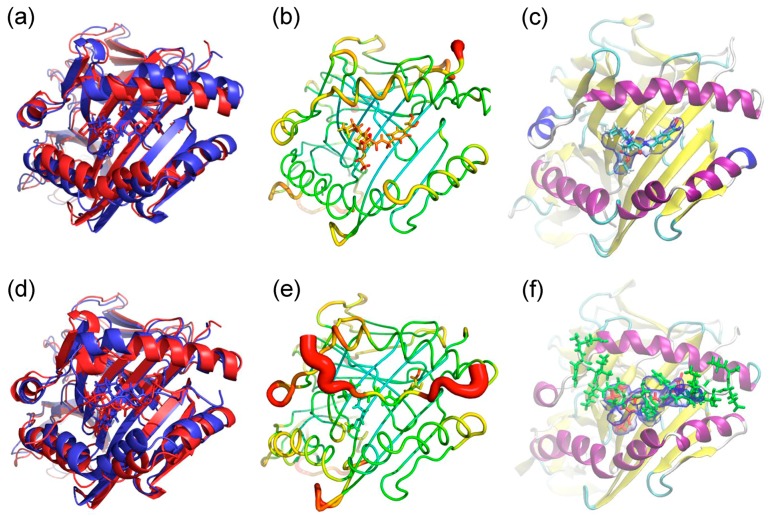
Simulated structures of ximelagatran-bound HLA-DRB1*07:01, in the absence (**a**–**c**) and presence (**d**–**f**) of the ligand peptide. (**a**,**d**) Alignment of the initial structure (red) and a representative structure (blue); (**b**,**e**) sausage plot of the structure where the color and the thickness of HLA are proportional to the RMSF of α carbon. The color scales for the sausage plots are the same; (**c**,**f**) The volume occupied by ximelagatran and the ligand peptide. The blue envelope outlines the 50% occupancy volume of ximelagatran (red) and the ligand peptide (green). α helix of HLA molecule is depicted in purple and β sheets in yellow.

MD simulations were performed to evaluate the possible effects of ximelagatran on the conformations of HLA-DRB1*07:01 and its ligand peptide. Each of the energies of the ximelagatran-bound simulations stabilized within about 0.5 ns (Figure 4a), indicating that the simulations were energetically stable. The flexibility of HLA was very similar in both simulations (Figure 4b). Figure 4c shows that the root mean square deviation (RMSD) as stabilizing after 1 ns to 2 ns in the absence of the ligand peptide (dimer), whereas it would rise in the presence of the ligand peptide (trimer), indicating that the ligand peptide would not find a stable conformation on top of ximelagatran. Figure 4d shows ximelagatran as keeping the peptide binding groove open. In Figure 5, structural analyses of the ximelagatran-bound simulations are presented in three panels. In the absence of the ligand peptide, the model shows ximelagatran as sprawling out across the P2–P6 pockets in the peptide binding groove and its methyl group as descending into the P4 pocket. This configuration is consistent with the EpiMatrix prediction of affinity for methyl moieties in leucine, methionine, and valine at this position. The simulated conformations of ximelagatran were similar in the absence and presence of the ligand peptide (Figure 5a,d), whereas the conformation was less mobile in a trimer simulation. Although ximelagatran showed to be mainly stationary in the presence of the ligand peptide, except for some flexibility at the end closest to the P6 pocket, the binding of N- and C-terminal portions of the ligand peptide appeared to be disrupted (Figure 5e). In the modeling some conformational changes were seen in the α and β chains, but the α chain helix was maintained.

### 2.3. Human Leukocyte Antigen (HLA) Class II Binding Assay and Liquid Chromatography Tandem Mass Spectrometry (LC-MS/MS) Detection of Ximelagatran Bound to HLA-DR Molecules

To reconfirm the inhibitory effect of ximelagatran on the binding of the ligand peptide to HLA-DRB1*07:01 [3], a DELFIA-based binding assay was conducted. As previously reported [3], ximelagatran inhibited the binding of the ligand peptide to HLA-DRB1*07:01, whereas no inhibitory effect was observed for HLA-DRB1*01:01 and DRB1*15:01 (Table 2). LC-MS/MS analysis of the same incubation samples revealed that the presence of HLA-DRB1*01:01 significantly increased the concentration of ximelagatran extracted from HLA class II binding assay samples (Table 3). Although the differences were not significant for HLA-DRB1*07:01 and DRB1*15:01, they appeared to be marginally significant (*p* = 0.087 and *p* = 0.084, respectively). Taken together, ximelagatran bound to all three HLA-DR molecules was detected in this setting. In contrast, the comparison between ximelagatran concentrations in (1) the presence of HLA-DR molecules and the ligand peptides; and (2) the presence of HLA-DR molecules and absence of the ligand peptides showed that the extent of the decrease of ximelagatran bound to the HLA-DR molecules by the addition of the ligand peptides was different between the three HLA-DR alleles (Table 3). The difference was significant (*p* < 0.05) for HLA-DRB1*01:01 and was marginally significant (*p* = 0.076) for DRB1*15:01, but just the overall trend for DRB1*07:01.

## 3. Discussion

In order to evaluate the possible direct interaction of ximelagatran with the peptide binding groove of HLA-DRB1*07:01, a series of in silico simulations and in vitro measurement of ximelagatran bound to HLA-DR molecules were performed.

The first docking studies indicated that ximelagatran and its active metabolite, melagatran, have high potential to interact with the peptide binding groove of HLA molecules compared with other IDT causing drugs, nevirapine [12] and allopurinol [13]. Ximelagatran showed higher binding affinities than melagatran to all three HLA-DR molecules. Considering that 40% to 70% of the oral dose of ximelagatran was absorbed in humans [14], the liver would be exposed to a high concentration of ximelagatran after oral administration, although it is rapidly metabolized (*t*_1/2_ = 0.34 h after oral administration) in humans [14]. Therefore, not melagatran (the predominant compound in human plasma), but rather ximelagatran is likely to be the primary compound responsible for ximelagatran-induced hepatotoxicity.

Additionally, MD simulations were conducted in order to see the possible effects of ximelagatran on the structure of the peptide binding groove of HLA-DRB1*07:01 and the conformation of bound ligand peptide, and to compare them with those of AdCaPy (one of the major histocompatibility complex loading enhancers [9]) and lapatinib [10]. The interaction modes of ximelagatran in the absence and presence of the ligand peptide were similar to those found in the docking study. Ximelagatran binds along the center of the peptide binding groove of HLA-DRB1*07:01 and makes its deepest contact in or near the P4 pocket. In the absence of the ligand peptide, ximelagatran keeps the peptide binding groove in an open state (Figure 4d and Figure 5a) similarly to AdCaPy, which enhances the loading efficiency of ligand peptides onto several HLA-DR molecules [9]. However, unlike AdCaPy, which is small and interacts with the P1 pocket of HLA-DR molecules, ximelagatran interacts along a relatively large part of the peptide binding groove of HLA-DRB1*07:01. As a result, the binding of the N- and C-terminal portions of the ligand peptide appears to be disrupted and, therefore, it does not seem to find a stable conformation on top of ximelagatran (Figure 5e). These results are consistent with the fact that ximelagatran functioned as a peptide competitor in vitro [3]. Although lapatinib is a much larger molecule and also interacts along a large part (P1–P6 pockets) of the peptide binding groove of HLA-DRB1*07:01, it could induce a tightly-closed binding groove structure that might be able to stabilize the binding of ligand peptides even in an irregular fashion [10]. MD simulations of ximelagatran and lapatinib indicated that small molecules can alter the antigen loading by affecting the conformation of bound peptides [6], as well as the structure of the peptide binding groove [10].

Finally, ximelagatran bound to three HLA-DR molecules was detected by LC-MS/MS in the absence of the ligand peptides (Table 3). This result was consistent with the docking study result in Table 1, which indicates the high potential of ximelagatran to interact with the peptide binding groove of these HLA-DR molecules. In contrast, this result seemingly sounds contradictory to the inhibitory effect of ximelagatran in vitro, which is HLA-DRB1*07:01 allele specific [3]. One possible reason might be seen in the comparison of the concentration of ximelagatran in the presence of HLA-DR molecules and the ligand peptides with that found in the presence of HLA-DR molecules and absence of the ligand peptides, in which the extent of the decrease of ximelagatran bound to the HLA-DR molecules by the addition of the ligand peptides was different between the three HLA-DR alleles. According to these results, one hypothesis could be assumed that ximelagatran interacts with a relatively broad range of HLA-DR alleles, but its affinity is not enough to affect the binding of their ligand peptides, and it is driven out from the peptide binding groove by the ligand peptides, except for in the case of HLA-DRB1*07:01. Ximelagatran was indeed simulated to bind to the peptide binding groove of each HLA-DR in a different manner (Figure 2), thus, it would be reasonable that its effects on the binding of ligand peptides are also different depending on the HLA alleles. For HLA-DRB1*07:01, the MD simulation indicated the possible disruption of the binding of the ligand peptide (Figure 5e), which is consistent with the in vitro results [3]. Similar MD simulations or docking studies with the ligand peptides for HLA-DRB1*01:01 and DRB1*15:01 are subjects for future study. Furthermore, this hypothesis might also be supported by the future optimization of the LC-MS/MS assay system whose current sensitivity might not be enough to detect a tiny amount of ximelagatran bound to HLA-DR molecules clearly. If the sensitivity of this assay system were optimized enough, it would be useful as a new IDT risk assessment system.

Taken together, these results indicate that ximelagatran directly interacts with the peptide binding groove of HLA-DRB1*07:01 and competes with ligand peptides for this binding site. In the cases of abacavir for HLA-B*57:01 [6,15], and lapatinib for HLA-DRB1*07:01 [10], in both of which drugs increase the loading of ligand peptides, it is easy to imagine the immune stimulation by the enhanced antigen presentation and/or the presentation of neo-antigens. Unlike these cases, it may be somewhat difficult to associate the inhibited antigen presentation by ximelagatran with the immune stimulation. However, considering the simulated binding mode of ximelagatran at the peptide binding groove of HLA-DRB1*07:01, there is a possibility that there are some kinds of peptides that are loaded onto HLA-DRB1*07:01 de novo and/or in a different manner in the presence of ximelagatran. Although further investigations are needed to reveal the conclusive mechanism, the present study strongly supports the interaction between ximelagatran and the peptide binding groove of HLA-DRB1*07:01, which could alter the immune response in some way and lead to the idiosyncratic ximelagatran-induced hepatotoxicity. In vitro studies using hepatocyte co-cultured with macrophages or lymphocytes carrying various HLA alleles will support our hypothesis and give us additional information to understand the detailed mechanism of ximelagatran-induced hepatotoxicity.

## 4. Materials and Methods

### 4.1. Docking Simulations

The crystal structures of HLA-DRB1*01:01 (PDB ID: 3PDO) and HLA-DRB1*15:01 (PDB ID: 1BX2) deposited at the Protein Data Bank [16] were used. As the crystal structure of HLA-DRB1*07:01 is not currently available, the 3D structure of HLA-DRB1*07:01 was constructed by homology modeling using the software HLA-Modeler (Ryoka Systems Inc., Tokyo, Japan) [17] and by employing the crystal structure of HLA-DRA1*01:01/DRB5*01:01 (PDB ID: 1H15) as the template structure. The binding modes and affinities of ximelagatran and its active metabolite, melagatran, at the peptide binding groove of three HLA-DR molecules were obtained by ASEDock docking simulations [18]. The binding affinities were judged by a scoring function of GBVI/WSA_dG, which is considered to express protein-ligand binding free energy [11]. A software system MOE (molecular operating environment) [19] was used. Considering the possible oxime-hydroxylamine tautomerization of ximelagatran, docking simulations were performed for both tautomers.

### 4.2. MD Simulations

MD simulations were carried out as described previously [10]. Briefly, each system was protonated at pH 7.4 and then solvated in a periodic rectangular box of TIP3P water with a 10 Å padding between the edge of the box and the nearest solute atom. Sodium ions were added to neutralize the system. The system was relaxed and then gradually heated to 300 K. The production simulations were performed in a canonical NVT ensemble, which simply ensured that the system maintained constant temperature and volume. An integration time step of 4 fs was used. The “representative structure” of each simulation was selected as the conformation that was the most representative of the conformations assumed over the final 1000 frames of the simulation [10]. A peptide derived from tetanus toxoid (TT 830-844; QYIKANSKFIGITEL) was used as the ligand peptide for HLA-DRB1*07:01. The structure of the TT peptide was obtained through homology modeling. Since there were no suitable PDB structures for the TT peptide, the peptide structure was created de novo using the Molefacture Protein Builder utility in the VMD software [20]. A series of peptide registration frames was considered as the starting position. The most likely peptide binding registration was selected according to the energetic stability, contact with α and β chains, and the EpiMatrix predicted registration. Based on these analyses, frame 3 was chosen for the TT peptide. The MD simulation of the ximelagatran-bound HLA molecule in the absence (dimer) and presence (trimer) of the TT peptide was 5 ns long.

### 4.3. HLA Class II Binding Assay and LC-MS/MS Detection of Ximelagatran Bound to HLA-DR Molecules

The binding assay was conducted as previously reported with minor modifications [21]. Briefly, soluble HLA-DR molecules (1600 ng/mL) provided by Benaroya Research Institute (Seattle, WA, USA) were incubated in quadruplicate with 1000 μM of ximelagatran and biotinylated control ligand peptides (influenza virus haemagglutinin 306–318: PRYVKQNTLKLAT for HLA-DRB1*01:01, TT 830-844 for HLA-DRB1*07:01 and myelin basic protein 94-112: NPVVHFFKNIVTPRTPPPS for HLA-DRB1*15:01) for 48 h at 37 °C to reach equilibrium. HLA-peptide complexes were then captured on a 96-well plate coated with L243 anti-HLA-DR antibody (BioLegend, San Diego, CA, USA, #307602). The plates were washed and incubated with Europium-labeled streptavidin (Perkin-Elmer, Waltham, MA, USA, #1244-360) for one hour at room temperature. The plates were washed again and DELFIA enhancement solution (Perkin-Elmer, #4001-0010) was added to develop the plates for 15 to 20 min at room temperature before they were read on an EnVision plate reader [21]. An aliquot of the incubation sample in the HLA class II binding assay was also captured on another L243 coated plate, and ximelagatran bound to HLA-DR molecules was extracted with 150 μL of acetonitrile. Concentrations of ximelagatran in extracted samples were determined by LC-MS/MS by a non-validated method using a Shim-pack XR-ODS (30 × 2 mm i.d., 2.2 μm) column. A Prominence LC-20A system (Shimadzu Corp., Kyoto, Japan) coupled with a Sciex API 4000 triple quadrupole mass spectrometer (Applied Biosystems, Foster City, CA, USA) was used. The aqueous mobile phase A was H_2_O/100 mM CH_3_CO_2_NH_4_/CH_3_CN (900/50/50, *v*/*v*/*v*) and the organic mobile phase B was CH_3_CN/100 mM CH_3_CO_2_NH_4_ (1000/50, *v*/*v*). The gradient was as follows: 50% B for the first 0.5 min, increased to 100% B from 0.5 s to 1 min, maintained at 100% B for 0.75 min. The total run time was 1.75 min with a flow rate set at 0.75 mL/min. The ionization was conducted in the positive ion mode using the transition *m*/*z* 474 to *m*/*z* 198. The injection volume was 10 µL. The retention time of ximelagatran was 1.2 min. The lower and upper limits of quantification of the assay were 0.1 and 100 nM, respectively.

### 4.4. Statistical Analysis

An unpaired *t*-test was carried out using Microsoft Excel 2010 (Microsoft Corp., Redmond, WA, USA) to compare the concentration of ximelagatran in the presence of HLA-DR molecules and absence of the ligand peptides with that of ximelagatran in the absence of HLA-DR molecules for each HLA-DR allele. When the binding of ximelagatran to HLA-DR molecules was indicated, for each HLA-DR allele an additional unpaired *t*-test was carried out where the concentration of ximelagatran in the presence of HLA-DR molecules and the ligand peptides was compared with that of ximelagatran in the presence of HLA-DR molecules and absence of the ligand peptides. A *p*-value of less than 0.05 was considered to be a significant difference.

## 5. Conclusions

A series of in silico simulations and in vitro measurement of ximelagatran bound to HLA-DR molecules indicated that ximelagatran directly interacts with the peptide binding groove of HLA-DRB1*07:01 and this interaction disrupts the binding of ligand peptides to this site, which could alter the antigen loading and lead to the idiosyncratic ximelagatran-induced hepatotoxicity.

## Figures and Tables

**Table 1:** The lowest generalized-Born volume integral/weighted surface area (GBVI/WSA_dG) values of the complexes between ximelagatran or melagatran and three human leukocyte antigen (HLA)-DR molecules.

HLA Allele	Compounds		
	Ximelagatran		Melagatran
	Tautomer	GBVI/WSA_dG (kcal/mol)	GBVI/WSA_dG (kcal/mol)
DRB1*01:01	Hydroxylamine	−11.88	−9.91
DRB1*07:01	Oxime	−11.24	−10.41
DRB1*15:01	Hydroxylamine	−10.99	−10.70

**Table 2:** The effect of ximelagatran on the binding of the ligand peptides to HLA-DR molecules. The effects of ximelagatran are expressed as a percentage of the binding of the ligand peptides compared with dimethyl sulfoxide (DMSO) control (*n* = 8). Values show average ± SD of quadruplicate.

HLA Allele	DRB1*01:01	DRB1*07:01	DRB1*15:01
% of DMSO control	105.7 ± 4.4	91.1 ± 13.4	112.3 ± 8.4

**Table 3:** Concentration of ximelagatran in the HLA class II binding assay samples detected by LC-MS/MS. Concentration of ximelagatran in each sample is expressed as average ± SD of quadruplicate. *p*-values were calculated for concentrations of ximelagatran in the absence of the ligand peptide compared with the absence of HLA-DR, and for concentrations of ximelagatran in the presence of HLA-DR and the ligand peptide compared with the presence of HLA-DR in the absence of the ligand peptide for each HLA-DR allele. (n/a = not applicable).

HLA Alleles	DRB1*01:01			DRB1*07:01			DRB1*15:01		
Incubation No.	1	2	3	4	5	6	7	8	9
HLA	−	+	+	−	+	+	−	+	+
Ligand peptide	+	−	+	+	−	+	+	−	+
Concentration (nM)	0.11 ± 0.01	0.17 ± 0.03	0.13 ± 0.01	0.13 ± 0.01	0.17 ± 0.03	0.14 ± 0.01	0.14 ± 0.03	0.23 ± 0.09	0.14 ± 0.02
*p*-Value	n/a	0.006 (vs. 1)	0.023 (vs. 2)	n/a	0.087 (vs. 4)	0.211 (vs. 5)	n/a	0.084 (vs. 7)	0.076 (vs. 8)

## Abbreviations

HLA	human leukocyte antigen
GBVI/WSA_dG	generalized-Born volume integral/weighted surface area
MD	molecular dynamics
LC-MS/MS	liquid chromatography tandem mass spectrometry
DMSO	dimethyl sulfoxide
IDT	idiosyncratic drug toxicity
ALT	alanine aminotransferase
RMSD	root mean-square-deviation
RMSF	root mean-square-fluctuation
TT	tetanus toxoid
